# Downregulation of HMGA1 Mediates Autophagy and Inhibits Migration and Invasion in Bladder Cancer via miRNA-221/TP53INP1/p-ERK Axis

**DOI:** 10.3389/fonc.2020.00589

**Published:** 2020-05-12

**Authors:** Xiaoqiang Liu, Zhengtao Zhou, Yibing Wang, Ke Zhu, Wen Deng, Yulei Li, Xiaochen Zhou, Luyao Chen, Yu Li, An Xie, Tao Zeng, Gongxian Wang, Bin Fu

**Affiliations:** ^1^Department of Urology, The First Affiliated Hospital of Nanchang University, Nanchang, China; ^2^Jiangxi Institute of Urology, Nanchang, China; ^3^Department of Emergency, The Second Affiliated Hospital of Nanchang University, Nanchang, China; ^4^Department of Urology, The People's Hospital of Jiangxi Province, Nanchang, China

**Keywords:** high-mobility group AT-hook 1 (HMGA1), bladder cancer, autophagy, miR-221, TP53INP1

## Abstract

MicroRNAs (miRNAs) have been implicated in regulating the development and metastasis of human cancers. MiR-221 is reported to be an oncogene in multiple cancers, including bladder cancer (BC). Deregulation of autophagy is associated with multiple human malignant cancers. Whether and how miR-221 regulates autophagy and how miR-221 has been regulated in BC are poorly understood. This study explored the potential functions and mechanisms of miR-221 in the autophagy and tumorigenesis of BC. We showed that the downregulation of miR-221 induces autophagy via increasing TP53INP1 (tumor protein p53 inducible nuclear protein 1) and inhibits migration and invasion of BC cells through suppressing activation of extracellular signal-regulated kinase (ERK). Furthermore, the expression of miR-221 is regulated by high-mobility group AT-hook 1 (HMGA1) which is overexpressed in BC. And both miR-221 and HMGA1 are correlated with poor patient survival in BC. Finally, the downregulation of HMGA1 suppressed the proliferative, migrative, and invasive property of BC by inducing toxic autophagy via miR-221/TP53INP1/p-ERK axis. Collectively, our findings demonstrate that the downregulation of miR-221 and HMGA1 mediates autophagy in BC, and both of them are valuable therapeutic targets for BC.

## Introduction

Bladder cancer (BC) is the fourth most common cancer in men in the United States with an estimated 62,380 new cases and 12,520 deaths in 2018 (1). Approximately 75% of cases are non-muscle-invasive BC (Ta, T1), which has a good prognosis, with 94% of patients surviving ≥5 years and is usually treated by transurethral resection and intravesical chemotherapy (2). However, approximately half of these patients will experience cancer recurrence, and 20% will progress to muscle-invasive BC (MIBC), which requires additional surgical interventions and chemotherapy (3). Although few patients are initially diagnosed with MIBCs, the 5-year survival of patients with MIBC is low: nearly 50.1% for regional stage and 10.2% for distant stage (4). Despite the crucial advances in BC research this year, the mortality rate of BC has remained unchanged due to the lack of specific targets (5).

Autophagy, which is a cellular self-degradative process mobilizing intracellular nutrient resources, plays an important role in cell survival under stress conditions. However, hyperactivated autophagy can lead to non-apoptotic programmed cell death (6). The deregulation of this process is associated with multiple human diseases, including cancer (7). In cancer, autophagy has a complex role and shows either an oncogenic or tumor suppressor activity (8). A feasible strategy to treat cancer is to reprogram cancerous cells to undergo toxic autophagy (9).

MicroRNAs (miRNAs) are an abundant class of single-stranded non-coding small RNAs that negatively regulate gene expression through inducing the degradation of target messenger RNAs (mRNAs) or translation repression by binding to the 3′ untranslated region (3′-UTR) of mRNAs (10). Emerging evidence indicates that the dysregulation of miRNA expression is involved in tumor initiation, proliferation, invasion, and metastasis (11). Thus, the identification of specific molecules that target miRNAs and its upstream transcription factors may be an effective approach to eradicate BC. MiR-221 has been frequently identified as deregulated across different cancer types, including BC (12). Nevertheless, the role of miR-221 in autophagy and the molecular mechanism of miR-221 being regulated in BC are still unclear.

High-mobility group A (HMGA) protein family consists of four members (HMGA1a, HMGA1b, HMGA1c, and HMGA2), which preferentially binds to the minor groove of AT-rich regions in double-stranded DNA and thereby modulates the transcription of several genes through direct protein-protein interactions with transcription factors by modifying its conformation and enhancing the affinity of its binding to DNA (13). They are encoded by two genes, *HMGA1* and *HMGA2*. *HMGA1* gene gives rise to three HMGA1 isoforms (HMGA1a, HMGA1b, and HMGA1c) through alternative splicing (14). HMGA1 has been reported to play an important role in many types of cancers, including lung cancer (15), colorectal cancer (16), breast cancer (17), cervical cancer (18), and BC (19). HMGA1 contributes to tumor formation and progression through several mechanisms: inactivation of the apoptotic function of p53 (20), enhancement of the expression of genes involved in stem cell and inflammatory pathway (21), and modulation of the expression of miRNAs and genes involved in cell cycle and epithelial-mesenthymal transition (13). However, the role of HMGA1 in BC remains unknown.

In this study, we investigated the role of miR-221 and HMGA1 and demonstrated that the downregulation of HMGA1 inhibits migration and invasion partly via miRNA-221/TP53INP1/p-ERK axis-mediated toxic autophagy in BC.

## Materials and Methods

### Cell Culture and Treatment

Human BC cells of the 5637, J82, EJ, UM-UC-3, and T24 lines, the SV-HUC-1 human immortalized uroepithelium cell line, and 293T cells were purchased from the Cell Bank of Type Culture Collection of Chinese Academy of Sciences, Shanghai Institute of Cell Biology (Shanghai, China). 5637, J82, and EJ cells were maintained in RPMI-1640 (Gibco; Thermo Fisher Scientific, Inc.). T24, UM-UC-3, and 293T cells were cultured in Dulbecco's modified Eagle's medium (DMEM; Gibco), and SV-HUC-1 cells were cultured in F-12K medium (Gibco). Media were supplemented with 10% heat-inactivated fetal bovine serum (FBS; Hyclone), 100 U/ml penicillin, and 100 μg/ml streptomycin at 37°C in a humidified incubator with 5% CO_2_.

### Luciferase Reporter Assay

The segments of wild-type (WT) TP53INP1 3′UTR and mutant (MUT) TP53INP1 3′UTR were PCR amplified and inserted into the Renilla luciferase gene (psi-CHECK-2 vector; Promega) to generate the TP53INP1 WT plasmids and TP53INP1 MUT plasmids. 5637 cells and 293T cells were transfected using Lipofectamine™ 2000 (Invitrogen) in accordance with the recommendations of the manufacturer. Luciferase vectors (100 ng) (TP53INP1 WT, TP53INP1 MUT, and the empty vectors) were individually transfected into 5637 cells and 293T along with 100 nM hsa-miR-221-3p mimics (RiboBio, Guangzhou, China) or 100 nM negative control (NC) (RiboBio, Guangzhou, China). At 48 h after transfection, luciferase assays were performed using the Dual-Luciferase Reporter Assay System (Promega). For each transfection, the luciferase activity of three replicates was considered.

### Lentiviral Vector Construction

Recombinant lentiviral expression plasmids (pLV-IRES-BSD-GFP-miR-221/pLV-IRES-BSD-GFP-sh-miR-221) and pLV-IRES-BSD-GFP-shTP53INP1 were constructed, and their identity was confirmed by DNA sequencing. To generate lentiviral particles, the recombinant expression plasmids were co-transfected with a packaging plasmid system into 293T cells, and the resultant viral particles were harvested at 48 h after transfection. 5637 cells were infected with the miR-221 lentiviral vector or with an NC vector, and T24 cells were infected with the sh-miR-221 lentiviral vector or with an NC vector, at a multiplicity of infection (MOI) of 50 and with polybrene (8 mg/ml) for 2 days. Infection efficiency was assessed in each experiment by observing the green fluorescent protein (GFP) expression under a fluorescence microscope.

### Transfection

Cells of the 5637 and T24 lines were plated in six-well plates at 2 × 10^5^ per well. The hsa-miR-221-3p mimic/inhibitor, NC, and HMGA1 siRNA were obtained from RiboBio (Guangzhou, China). The sequences of miR-221-3p mimics and miR-221-3p inhibitor were 5′-AGCUACAUUGUCUGCUGGGUUUC-3′ and 5′-GAAACCCAGCAGACAAUGUAGCU-3′, respectively. The following siRNA sequences were used: HMGA1 siRNA (5′-GGACAAGGCUAACAUCCCATT-3′), ATG5 siRNA (5′-GGATGAGATAACTGAAAG-3′), and NC siRNA (5′-UUCUCCGAACGUGUCACGUTT-3′). StarBase v2.0 (http://starbase.sysu.edu.cn/) was used to identify the predicted target of miR-221-3p. 5637 and T24 cells grown to 70–80% confluence were transfected using the Lipofectamine™ 2000 transfection reagent (Invitrogen, USA) and Opti-MEM medium (Gibco) in accordance with the instructions of the manufacturer. Six hours after transfection, the medium was replaced with fresh medium containing 10% FBS.

### RNA Extraction and Real-Time Quantitative PCR Analysis

Total RNAs were extracted from BC cell lines (5637, T24, J82, EJ, UM-UC-3) and SV-HUC-1 human immortalized uroepithelium cell line transfected with or without miRNA mimic/inhibitor or siRNA using TRIzol reagent (Invitrogen). cDNA was synthesized using a Takara PrimeScript RT reagent Kit (Takara) and specific miR-221 primers from the Bulge-Loop™ hsa-miR-221-3p RT-qPCR Primer Set (RiboBio, Guangzhou, China) for quantitative miRNA analysis. Quantitative real-time PCR was conducted on the ABI PRISM 7500 real-time PCR system (Applied Biosystems, USA). Real-time PCR results were normalized against an internal control U6. Relative expression levels were evaluated using the 2^−ΔΔCt^ method and then expressed as fold changes. For RT-qPCR, 400 ng of total RNA was used in a Takara PrimeScript RT reagent Kit (Takara) in accordance with the manufacturer's instructions, and PCR was performed on the ABI PRISM 7500 real-time PCR system (Applied Biosystems). The expression level of glyceraldehyde-3-phosphate dehydrogenase (GAPDH) was used as the internal control. All reactions were performed at least in triplicate. The oligonucleotide sequences of the RT-qPCR primers for *HMGA1, TP53INP1*, and *GAPDH* were as follows: HMGA1-forward, 5′-GCTGGTAGGGAGTCAGAAGGA-3′, and HMGA1-reverse, 5′-TGGTGGTTTTCCGGGTCTTG-3′; TP53INP1-forward, 5′-GACTTCATAGATACTTGCAC-3′, and TP53INP1-reverse, 5′-ATTGGACATGACTCAAACTG-3′; GAPDH-forward, 5′-CATGAGAAGTATGACAACAGCCT-3′, and GAPDH-reverse, 5′- AGTCCTTCCACGATACCAAAGT-3′.

### Western Blot Analysis

Total proteins from cells were prepared using radioimmunoprecipitation assay (RIPA) lysis buffer (Beyotime, Jiangsu, China). Protein was quantified using a Pierce BCA Protein Assay Kit (Thermo Fisher Scientific), followed by Western blot analysis. Once transferred to a polyvinylidene fluoride (PVDF) membrane, antibodies targeting GAPDH (Abcam, ab8245, 1:1,000), TP53INP1 (Abcam, ab32131, 1:1,000), HMGA1 (Abcam, ab32131, 1:1,000), ERK1/2 (CST, 3195, 1:1,000), p-ERK1/2 (CST, 9523, 1:1,000), LC3B (CST, 79424, 1:1,000), poly (ADP-ribose) polymerase (PARP, CST, 9532, 1:1,000), Beclin1 (CST, 3495, 1:1,000), ATG5 (CST, 12994, 1:1,000), and p62 (CST, 79424, 1:1,000) were used with a secondary antibody horseradish peroxidase-labeled goat anti-rabbit (1:4,000; 1.5 h of incubation at room temperature; Abcam, ab6721). GAPDH served as an internal control. Protein bands were visualized by enhanced chemiluminescence.

### Cell Viability Assay

The effects of siHMGA1 on the viability of T24 cells were assessed using a Cell Counting Kit-8 (CCK-8) detection kit (Nanjing Keygen Biotech Co., Ltd., Nanjing, China) as described in Liu et al. (22). T24 cells were transfected with siHMGA1 and NC siRNA as described before. The cells were incubated at 37°C for 24, 48, 72, and 96 h. Absorbance was measured at 450 nm. Cell viability was expressed as a percentage of absorbance in the siHMGA1-transfected wells compared with that of the NC siRNA wells. Each experiment was performed in triplicate and repeated at least three times.

### Colony Formation Assay

To perform the colony formation assays, the cells were transfected with siHMGA1 and/or miR-221-3p mimic as described before. After 24 h of transfection, the cells were trypsinized, counted, and replated to the six-well plate in equal numbers (200 cells). After 9–13 days of culture, the cells were fixed in methanol for 30 min, stained with crystal violet (C3886, Sigma-Aldrich) for 30 min, washed several times with phosphate buffered saline (PBS), and then photographed.

### Cell Migration and Transwell Assay

Cell migration was determined using a wound healing assay. 5637 and T24 cells were seeded in six-well plates and transfected with pLV-IRES-BSD-GFP-miR-221/pLV-IRES-BSD-GFP-sh-miR-221, HMGA1 siRNA, or non-target control for 48 h. A wound was created by scratching the monolayer with a 200-μl pipette tip. The wounded monolayer was then washed three times with PBS to remove cell debris. After scratching, the area of the cell-free scratch was photographed with Olympus CKX41 inverse microscope at 0 and 24 h. Cell invasion capacities were measured by a transwell assay (Corning, Toledo, OH, USA). 5637 and T24 cells were transfected with pLV-IRES-BSD-GFP-miR-221/pLV-IRES-BSD-GFP-sh-miR-221, miR-221 mimic, HMGA1 siRNA, or non-target control for 48 h and then seeded in the upper chamber of the transwell system. The upper chamber of the insert was precoated with 0.1 ml (300 μg/ml) of Matrigel matrix (Corning) for the invasion assay. Invaded cells on the underside of the membrane were counted. In this assay, prepared cells were seeded in the upper chamber with a serum-free medium, and the medium of the lower chamber was supplemented with 10% FBS as a chemoattractant. After 24 h of incubation, the cells were fixed with 4% formaldehyde. Cells that did not invade through the pores were removed with a cotton swab. Cells that had migrated or invaded the lower surface of the membrane were stained with crystal violet. Finally, five representative fields for each well were randomly imaged at 100× magnification and quantified using Olympus CKX41 inverse microscope.

### Tissue Microarray and Immunohistochemistry

Human BC tissue microarrays (TMAs) containing 48 pairs of tumors and matched adjacent tissues and 16 tumors were purchased from Xi'an Alenabio Biotech Co., Ltd. (Xi'an, China). The immunohistochemical staining of TMAs using primary antibodies against HMGA1 (Abcam, ab32131) was performed as previously described (23). Briefly, after dewaxing, rehydration, antigen retrieval, and blocking, the arrays were incubated overnight at 4°C with HMGA1 antibody. Color detection was performed by 3,3′-diaminobenzidine (DAB) and hematoxylin counterstaining. Stained tumor and adjacent normal tissues were classified into two groups (low and high) in accordance with the staining intensity of each tissue.

### Autophagic Vacuoles Staining

Autophagosome formation was analyzed using a Cyto-ID autophagy detection kit (Enzo Life Sciences) according to the manufacturer's instruction (24) and LC3 antibody staining. Briefly, for Cyto-ID autophagy detection kit staining, cells were stained with Cyto-ID autophagy detection dye and Hoechst 33342 for 30 min at 37°C. For LC3 antibody staining, cells were fixed with methanol for 10 min and blocked with 2.5% BSA for 30 min, incubated with an anti-LC3B antibody (1:200; CST) at 4°C overnight, washed three times with PBS, and incubated with an Alexa Fluor 488-conjugated secondary antibody (1:500; Invitrogen) for 1 h. Slides were imaged under a microscope (Nikon).

### Statistical Analysis

All data were presented as the means ± SD in at least three replicates per group. Statistical analysis was performed to determine the significance of the difference between groups using ANOVA or Student's *t-*test. All statistical analyses were performed using GraphPad/Prism software for Windows. *p* < 0.05 was considered statistically significant.

## Results

### Downregulation of miR-221 Promotes Autophagy in Bladder Cancer Cells

**Figure 1:**
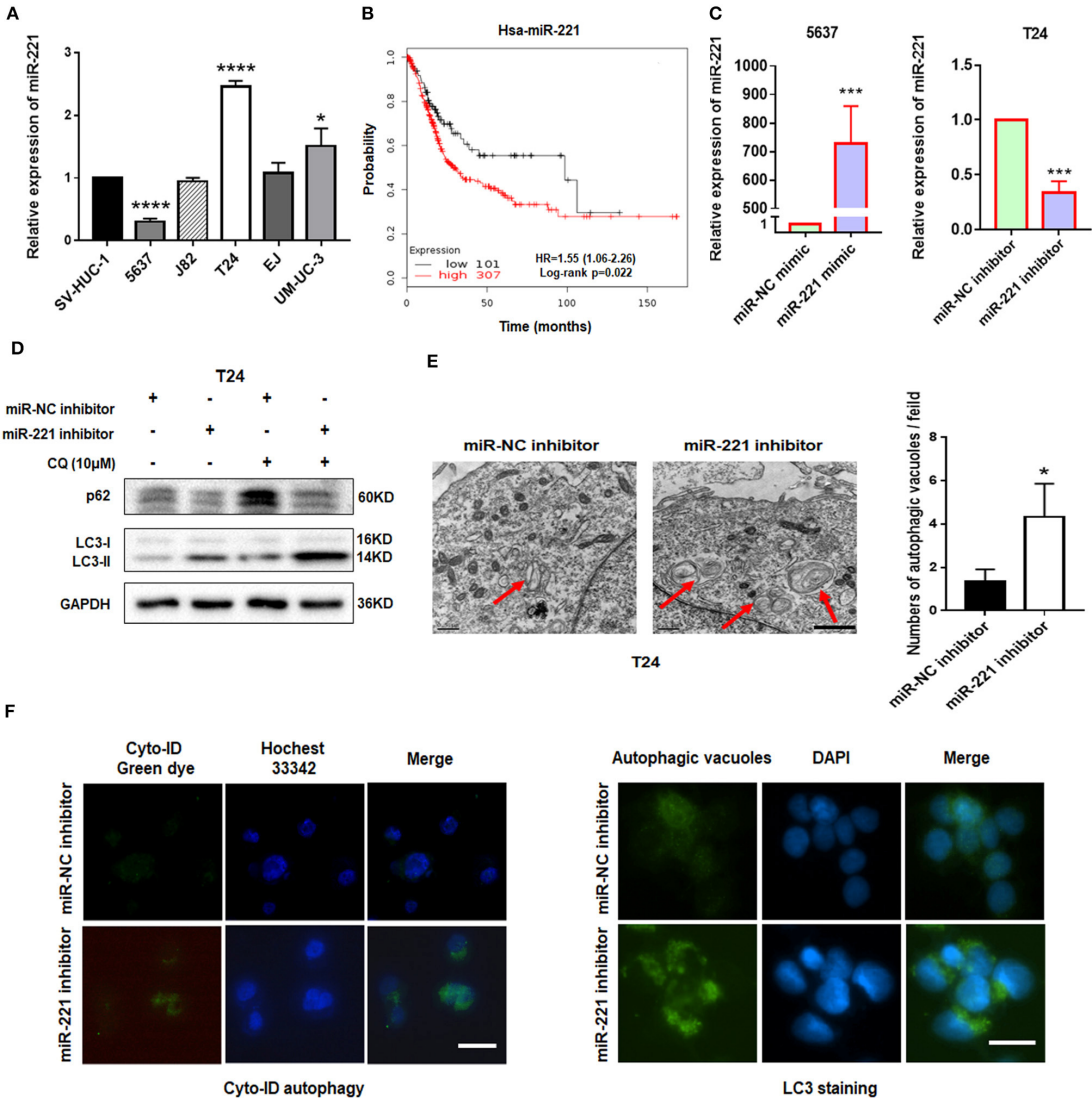
Downregulation of miR-221 induces autophagy in bladder cancer (BC) cells. **(A)** The expression of miR-221 was determined by RT-qPCR in BC cell lines. SV-HUC-1 was used as a normal control. **(B)** The Kaplan–Meier survival analysis of low and high level of miR-221 from The Cancer Genome Atlas (TCGA) database. **(C)** Relative expression of miR-221 after knockdown and overexpression of miR-221 examined by RT-qPCR. **(D)** T24 cells were transfected with miR-221 inhibitor or miR-NC inhibitor for 24 h, followed by exposure to 10 mM chloroquine (CQ). **(E)** Two representative transmission electron microscopy (TEM) images were identified to show the autophagosome after knockdown of miR-221 for 24 h. Red arrows indicate autolysosome. Scale bar, 1 μm. **(F)** T24 cells were transfected with miR-221 inhibitor for 48 h and then stained with Cyto-ID Autophagy Detection Kit (left) and LC3 antibody (right). Scale bar, 50 μm. *p < 0.05, ***p < 0.001, and ****p < 0.0001.

Our previous study showed that miR-221 mediates the immune evasion of BC cells (25). However, the molecular mechanism of miR-221 action in BC remains unclear. To further investigate the role of miR-221 in BC cells, we first examined the expression level of miR-221 in a panel of five BC cell lines (5637, J82, T24, EJ, and UM-UC-3) and the human immortalized uroepithelium cell line (SV-HUC-1). We showed that miR-221 was significantly upregulated or unchanged in invasive BC lines (T24, J82, EJ, UM-UC-3) but downregulated in the non-invasive BC cell line (5637) (Figure 1A), which indicated that the overexpression of miR-221 might be specifically correlated to MIBC. Moreover, we performed Kaplan–Meier analysis in the database (http://kmplot.com/analysis/) and found that patients with BC with high miR-221 expression had worse prognosis and shorter survival time than those with low miR-221 expression (Figure 1B).

Autophagy is a vital regulator of invasion and migration in cancer (26). Previous studies have revealed that miR-221 inhibits autophagy and promotes heart failure by modulating the p27/CDK2/mTOR axis and inhibits hypoxia/reoxygenation-induced autophagy through the DDIT4/mTORC1 and TP53INP1/p62 pathways (27, 28). Given that our previous study showed that the downregulation of miR-221 inhibits the migration and invasion of BC cells, we investigated whether miR-221 could influence autophagy of BC cells. We transfected the non-invasive BC cell line 5637 and invasive BC cell line T24 with miR-221 mimic and miR-221 inhibitor and verified the expression of miR-221 by RT-qPCR (Figure 1C). Immunoblotting analysis showed that the downregulation of miR-221 increased the LC3-II/LC3-I expression ratio and decreased the p62 expression in T24 cells. And lysosomal inhibitor chloroquine (CQ) could further promote LC3-II accumulation compared to miR-221 inhibitor action alone (Figure 1D). We further analyzed the ultrastructure of T24 BC cells by using transmission electron microscopy (TEM). Representative TEM images are shown in Figure 1E. Typical autolysosomes were observed in T24 cells, whereas the few autolysosomes were observed in control cells after transfection with the miR-221 inhibitor. Moreover, we detected autophagic flux by using Cyto-ID staining and LC3 antibody staining as reported (29, 30). As presented in Figure 1F, we found that the suppression of miR-221 expression induced the formation and accumulation of autophagosomes (green fluorescence) in T24 cells (Figure 1F). Thus, our results indicated that the suppression of miR-221 expression promoted autophagy in BC cells.

### Downregulation of miR-221 Induces Autophagy and Inhibits Invasion and Migration of Bladder Cancer Cells *in vitro* via TP53INP1/p-ERK Axis

**Figure 2:**
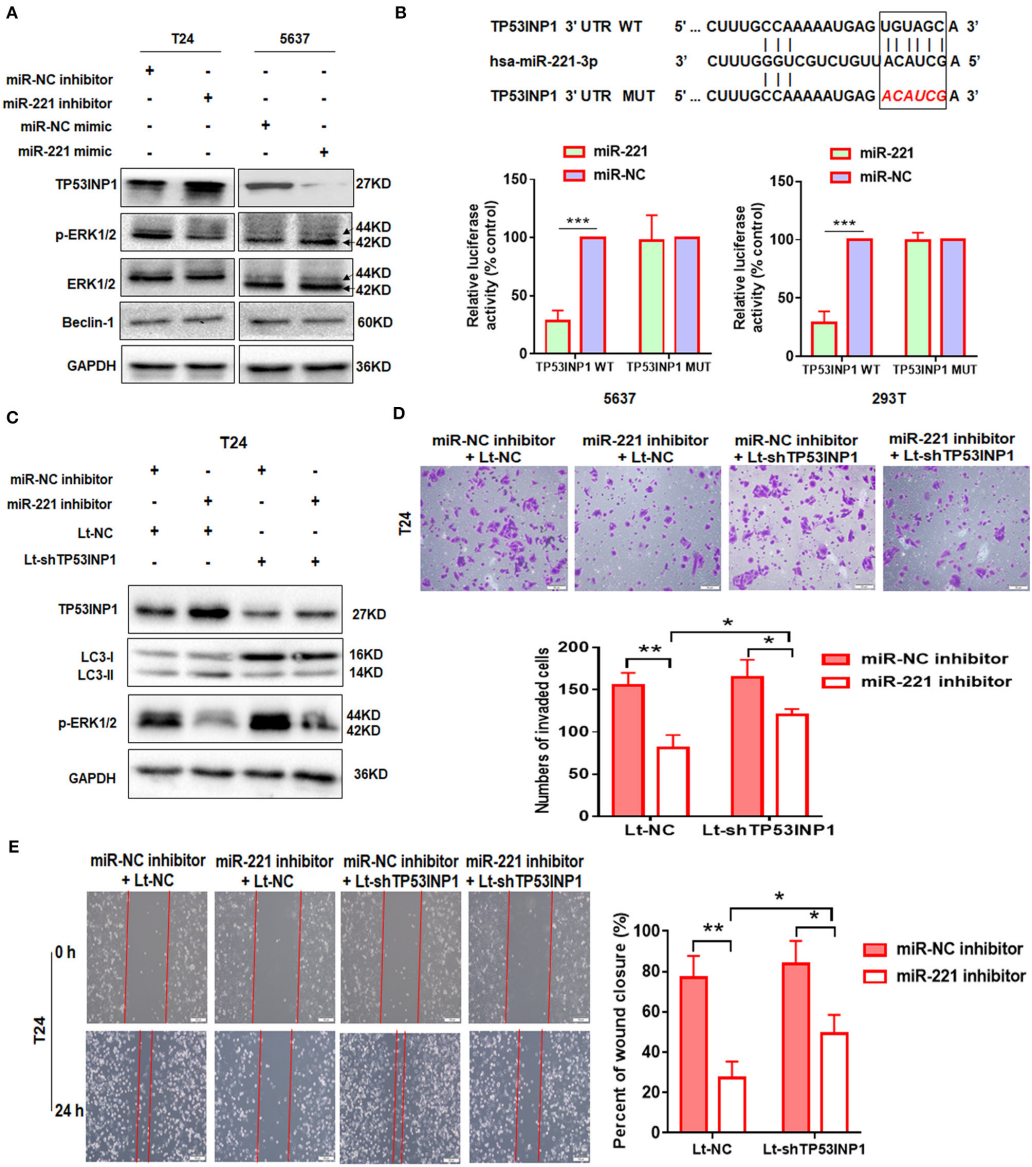
TP53INP1 is the direct target of miR-221. **(A)** The protein expression of p62, LC3, TP53INP1, p-ERK1/2, and ERK1/2 in T24 cells treated by miR-221 inhibitor and 5637 cells treated by miR-221 mimic. Glyceraldehyde-3-phosphate dehydrogenase (GAPDH) was used for normalization. **(B)** Schematic illustration of the sequence of hsa-miR-221 and its complementary sequence in 3′UTR of TP53INP1 mRNA, in which the letters in red represent the five nucleotides we mutated. Luciferase activity assays using luciferase reporters with wild-type TP53INP1 3′UTR (TP53INP1 WT) or mutant TP53INP1 3′UTR (TP53INP1 MUT) were performed with co-transfection of miR-221 mimics or negative control into 5637 bladder cancer (BC) cells and 293T cells. Data represent the mean ± SD of triplicates. **(C–E)** T24 cells were transfected with miR-221 inhibitor or Lt-TP53INP1 for 48 h. A portion of the cells were harvested for IB analysis **(C)**. The other portion of cells were seeded in the upper chamber of the transwell system **(D)** or plated into six-well plates for 24 h, followed by wound healing assay **(E)**. Scale bar, 50 μm **(D)**, 100 μm **(E)**. *p < 0.05, **p < 0.01, and ***p < 0.001.

To further illustrate the mechanism underlying the autophagy-regulating effect of miR-221, we screened for the candidate autophagy-related targets of miR-221 using StarBase v2.0 (http://starbase.sysu.edu.cn/) and literature search. MiR-221 inhibits hypoxia/reoxygenation-induced autophagy through TP53INP1/p62 pathways (28). Thus, we speculated that miR-221 could modulate autophagy and then regulate the invasion and migration of BC cells by targeting TP53INP1. Interestingly, consistent with this prediction, miR-221 suppressed TP53INP1 expression in 5637 cells, and the suppression of miR-221 expression increased the expression of TP53INP1 in T24 cells (Figure 2A). Although Beclin1 was identified as a target of miR-221, we did not detect a significant change in the expression of BC cells transfected with the miR-221 mimic or inhibitor. Previous studies have revealed that the downregulation of TP53INP1 could activate a p73-dependent DUSP10/ERK signaling pathway to promote the metastasis of hepatocellular carcinoma (31). Western blot analyses showed that the phosphorylation of ERK1/2 was significantly repressed after suppressing the expression of miR-221, and the expression of total ERK1/2 was unchanged in T24 cells (Figure 2A).

We constructed a luciferase reporter to further validate whether miR-221 directly targets the 3′UTR of TP53INP1. The predicted binding sites of TP53INP1 are shown in Figure 2B. Luciferase reporter assays confirmed that miR-221 could reduce the luciferase activity in the cells transfected with the plasmid harboring WT *TP53INP1* 3′UTR (*TP53INP1* WT) but did not affect luciferase activity in the cells transfected with plasmids harboring mutant *TP53INP1* (*TP53INP1* MUT) (Figure 2B).

Next, we determined whether suppression of miR-221 promoted autophagy and inhibited invasion and migration of BC cells via increasing TP53INP1. We found that the downregulation of miR-221-induced autophagy could be partially blocked by knockdown of TP53INP1 (Figure 2C). Furthermore, the downregulation of miR-221-induced decrease of ERK phosphorylation was largely blocked by TP53INP1 knockdown (Figure 2C). On the other hand, the inhibition of invasion and migration induced by miR-221 suppression could be partly blocked by TP53INP1 knockdown (Figures 2D,E). Taken together, the downregulation of miR-221 could induce autophagy and inhibit invasion and migration of BC cells *in vitro* through the TP53INP1/p-ERK axis.

### The Oncogenic Effect of miR-221 Is Extracellular Signal-Regulated Kinase Pathway-Dependent, and miR-221 Suppression-Induced Inhibition of Invasion and Migration Is Autophagy-Dependent

**Figure 3:**
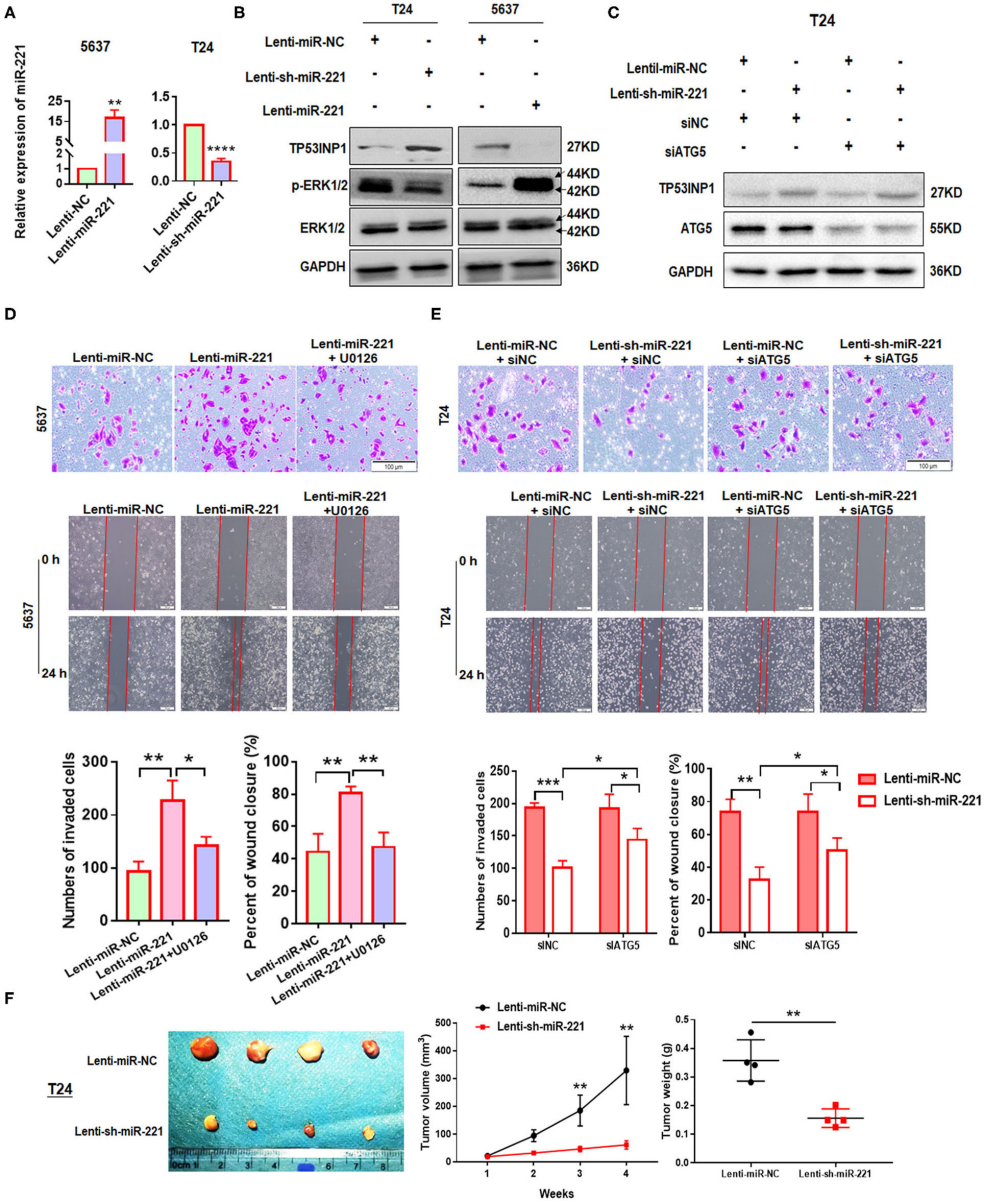
MiR-221 suppression-induced inhibition of invasion and migration is autophagy dependent. **(A)** Relative expression of miR-221 after overexpression and knockdown of miR-221 in 5637 and T24 cells detected by RT-qPCR. **(B)** Validation of the expression of TP53INP1, p-ERK1/2, and ERK1/2 using Western blot analysis. Glyceraldehyde-3-phosphate dehydrogenase (GAPDH) was detected as loading control. **(C)** T24 cells were transfected with Lenti-sh-miR-221 or siRNA targeting ATG5 (siATG5). **(D,E)** Transwell invasion assays and wound healing assay of 5637 cells treated with Lenti-miR-221 and ERK inhibitor U0126 and T24 cells treated with Lenti-sh-miR-221 or siRNA targeting ATG5. Scale bar, 100 μm. **(F)** For the *in vivo* analyses, 5 × 10^6^ T24-sh-miR-221 cells were injected subcutaneously into the posterior hip of nude mice. The mice were continuously observed for 30 days. Images of the tumors generated by T24-sh-miR-221 cells (*n* = 4). Tumor volume was monitored every week. Tumor weight was evaluated in T24-sh-miR-221 or miR-NC treated mice. *p < 0.05, **p < 0.01, ***p < 0.001, and ****p < 0.0001.

To further investigate the role of miR-221 in BC, we transfected the non-invasive BC line 5637 with a lentiviral vector encoding the human *MIR221* gene, while invasive BC cell line T24 was transfected with a specific *MIR221*-shRNA lentivirus. The transfection efficiency and expression of miR-221 were verified by RT-qPCR (Figure 3A). Subsequent Western blot analysis showed that the ectopic overexpression of miR-221 decreased TP53INP1 expression and enhanced the phosphorylation of ERK1/2 in 5637 cells. By contrast, the suppression of endogenous miR-221 expression increased the expression of TP53INP1 and repressed the phosphorylation of ERK1/2 in T24 cells (Figure 3B). Transwell and wound-healing assays showed that the ectopic overexpression of miR-221 could remarkably promote the invasive and migratory capacity of 5637 BC cells (Figure 3D), whereas the suppression of endogenous miR-221 expression could impede the invasive and migratory capacity of T24 BC cells (Figure 3E). To further validate the role of p-ERK1/2 signaling in miR-221-regulated invasion and migration of BC cells, we analyzed the impact of introducing an ERK inhibitor (U0126) into 5637 cells with stable miR-221 overexpression. As presented in Figure 3D, the introduction of U0126 attenuated *in vitro* cell migration and invasion abilities, suggesting that ERK signaling is needed to drive invasion and migration in BC cells with ectopic miR-221 overexpression.

Furthermore, to investigate whether the effects of miR-221 suppression on cellular invasion and migration were due to autophagy induction, we determined the effect of ATG5 knockdown (Figure 3C). We found that ATG5 knockdown partly blocked miR-221 suppression-induced inhibition of invasion and migration (Figure 3E).

Thereafter, we determine the impact of miR-221 on BC progression *in vivo*. We established a xenograft model by the subcutaneous implantation of T24-sh-miR-221 cells and their control cells into the posterior hip of nude mice, which were killed after 30 days. We found that the implantation of sh-miR-221 cells significantly impacted the BC cell proliferation *in vivo* (Figure 3F). Overall, these results suggested that the oncogenic effect of miR-221 is ERK pathway-dependent, and the effects of miR-221 suppression on cell invasion and migration are autophagy-dependent.

### High-Mobility Group AT-Hook 1 Was Upregulated in Bladder Cancer and Correlated With miR-221

**Figure 4:**
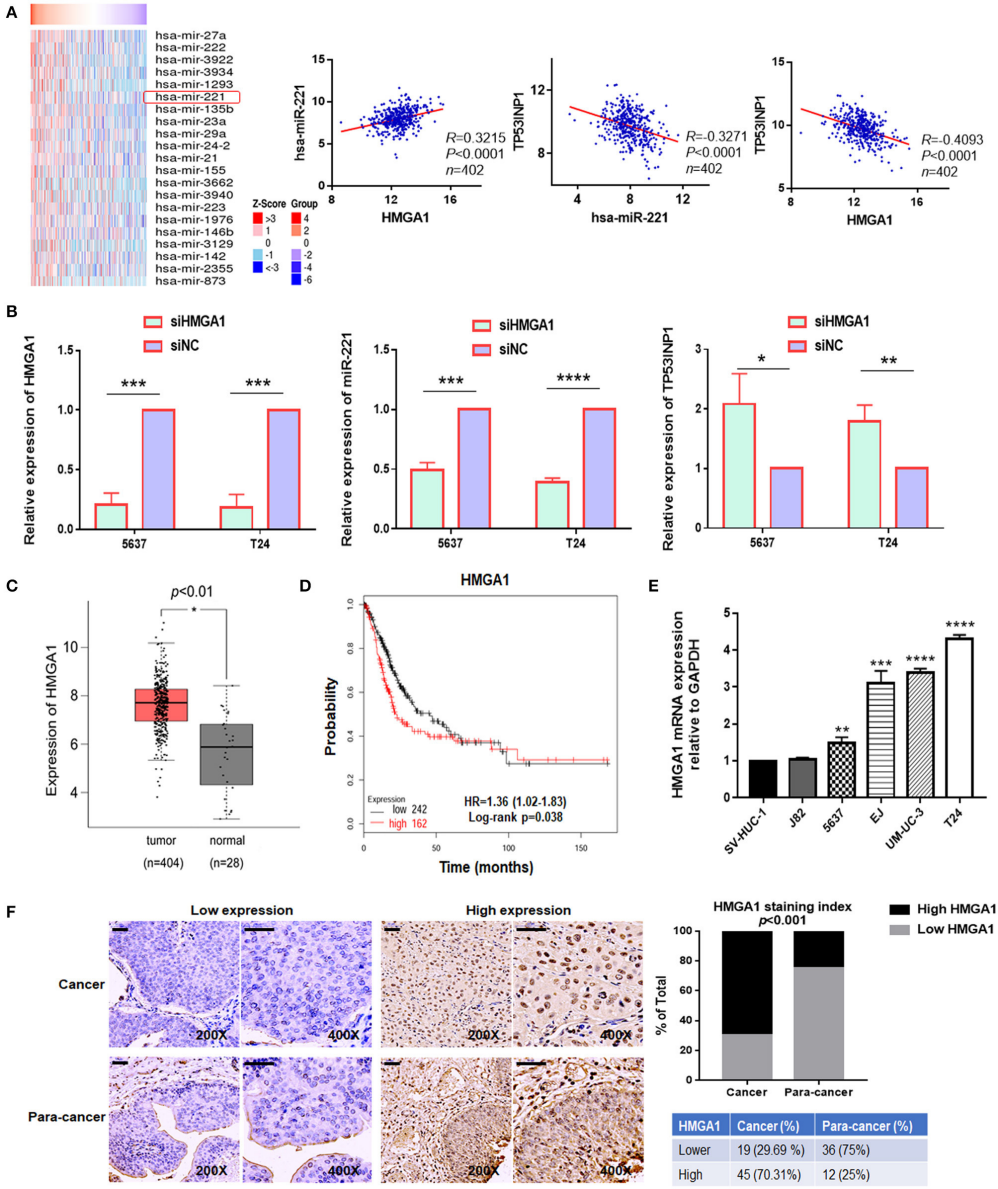
The expression of high-mobility group AT-hook 1 (HMGA1) and its correlation with miR-221 in bladder cancer (BC). **(A)** Heatmap diagram of differential miRNAs which were positively correlated with HMGA1 in The Cancer Genome Atlas (TCGA) database (left). The correlation between HMGA1 and miR-221, miR-221 and TP53INP1, and HMGA1 and TP53INP1 were examined in 402 cases of BC samples in the TCGA database (right). **(B)** The effects of siHMGA1 on the expression of HMGA1, miR-221, and TP53INP1 in 5637 and T24 cells were determined by RT-qPCR. **(C)** Expression of HMGA1 in BC samples (red box) and adjacent tissues (gray box). **(D)** The Kaplan–Meier survival analysis of low and high level of HMGA1 from the TCGA database. **(E)** The expression of HMGA1 was determined by RT-qPCR in BC cell lines. SV-HUC-1 was used as a normal control. **(F)** Immunohistochemistry (IHC) staining of HMGA1 in 64 BC tissues and paired 48 adjacent non-tumor tissues obtained from patients with BC. Scale bar, 50 μm. *p < 0.05, **p < 0.01, ***p < 0.001, and ****p < 0.0001.

Thereafter, we investigated how miR-221 was upregulated in invasive BC cells. Previously, miR-221 is targeted and regulated by transcriptional factor HMGA1 during cervical cancer (18). In addition, as a key regulator of the autophagic pathway in cancer cells, HMGA1 might contribute to cancer progression (6). And HMGA1 mRNA was upregulated in BC and correlated with the prognosis of BC patients (19). However, the role of HMGA1 in BC remains unclear. It was unknown whether the miR-221-mediated autophagy, migration, and invasion of BC cells were regulated by HMGA1. We further evaluated the correlation between HMGA1 and miR-221 in The Cancer Genome Atlas (TCGA) database by LinkedOmics (32), in which we observed a strong and positive correlation between miR-221 and HMGA1 and a negative correlation between miR-221 and TP53INP1, HMGA1, and TP53INP1 in 402 BC tissues (Figure 4A). Given that HMGA1 might act as upstream of miR-221 and was overexpressed in BC, HMGA1 expression was silenced by siRNA transfection in the human BC T24 and 5637 cells (Figure 4B). We first evaluated the effect of HMGA1 silencing on the expression of miR-221 and TP53INP1 by RT-qPCR, observing a strong reduction of miR-221 and an increase of TP53INP1 (Figure 4B). Altogether, these data suggest that HMGA1 is correlated with miR-221 in BC.

We first chose to evaluate the expression of HMGA1 in TCGA database by Gene Expression Profiling Interactive Analysis (GEPIA) for exploring the function of HMGA1 in the progression of BC (33). Notably, HMGA1 expression was significantly increased in BC tissues and cell lines (Figures 4C,E). Furthermore, consistent with a previous study, the present study found that the high expression of HMGA1 was correlated with poor prognosis of patients with BC as revealed by provisional TCGA database analysis in the database (http://kmplot.com/analysis/) (Figure 4D). Next, we tested the HMGA1 level in 64 BC tissues and paired 48 adjacent non-tumor tissues by immunohistochemistry. As presented in Figure 4F, HMGA1 was highly expressed in 45/64 (70.31%) BC and 12/48 (25%) paracancer tissues (*p* < 0.001).

### Downregulation of High-Mobility Group AT-Hook 1 Inhibits the Proliferation, Colony Formation, Invasion, and Migration of Bladder Cancer Cells by Promoting Autophagy

**Figure 5:**
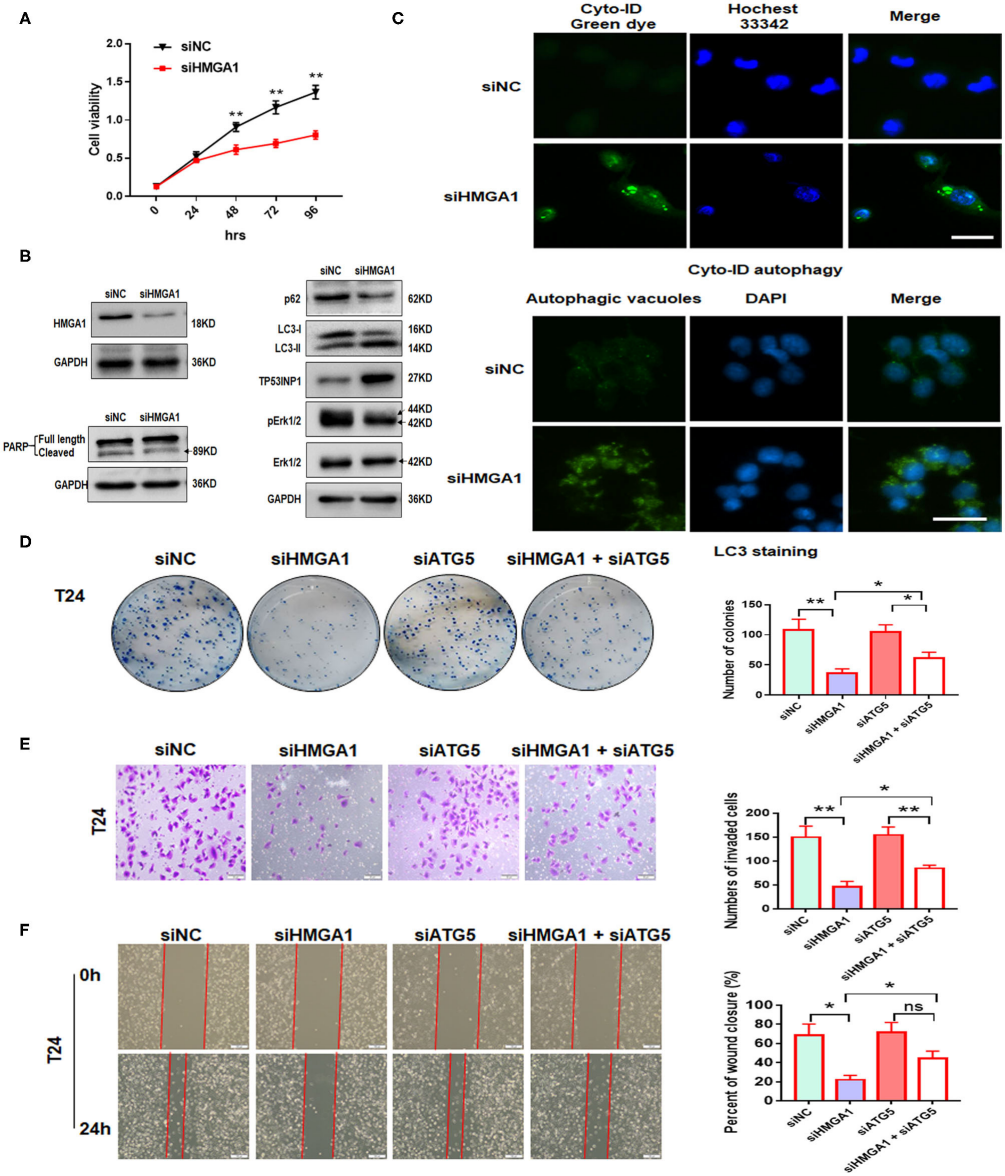
The effect of high-mobility group AT-hook 1 (HMGA1) knockdown on autophagy and growth, invasion, and migration of bladder cancer (BC) cells. **(A)** Cell Counting Kit-8 (CCK-8) cell viability assay of T24 cells treated with siHMGA1. **(B)** The protein expression of HMGA1, poly (ADP-ribose) polymerase (PARP), p62, LC3, TP53INP1, p-ERK1/2, and ERK1/2 in T24 cells transfected with HMGA1 siRNA. **(C)** T24 cells were transfected with HMGA1 siRNA for 48 h and then stained with Cyto-ID Autophagy Detection Kit and LC3 antibody. Scale bar, 50 μm. **(D)** Cell clone numbers were counted when HMGA1 or ATG5 was silenced in T24. **(E)** The effect of HMGA1 or ATG5 knockdown on the invasive ability of T24 cells as detected by transwell invasion assay. Scale bar, 50 μm. **(F)** The effect of HMGA1 or ATG5 silencing on cell migration of T24 cells performed by wound healing assay. Scale bar, 100 μm. *p < 0.05, **p < 0.01 and ns, no statistical significance.

Next, we investigated the effect of HMGA1 on the proliferation of BC cell lines. CCK8 assays showed that HMGA1 silencing inhibited the proliferative capacities of T24 cells (Figure 5A). Consistent with the abovementioned results, the colony formation assays showed that the suppression of HMGA1 decreased colony formation (Figure 5D), and the transwell invasion and wound healing assays indicated that HMGA1 silencing significantly inhibited cell migration and invasion (Figures 5E,F).

To further illustrate the molecular mechanisms underlying the downregulation of the HMGA1-induced inhibition on BC cell proliferation, invasion, and migration, autophagy and apoptosis-related proteins were examined through Western blot analysis after HMGA1 knockdown in BC cells. We silenced HMGA1 expression in T24 cells by transfecting T24 cells with HMGA1 siRNA and examined the HMGA1 protein expression by Western blot analysis. As expected, the HMGA1 protein level was strikingly reduced in the siHMGA1 group compared with that in the siNC group. Consistent with a previous study (6), HMGA1 silencing significantly increased LC3-II protein levels and reduced p62 protein levels but did not induce apoptosis in T24 cells, as assessed by measuring the cleavage of PARP (Figure 5B). Interestingly, HMGA1 knockdown increased TP53INP1 expression and repressed the phosphorylation of ERK1/2 in T24 cells (Figure 5B). In addition, HMGA1 knockdown induced the formation and accumulation of autophagosomes (green fluorescence) in T24 cells (Figure 5C).

Furthermore, to investigate whether the effects of HMGA1 knockdown on cellular proliferation, invasion, and migration were due to autophagy induction, we knocked down ATG5 to abrogate autophagy. We found that ATG5 knockdown partly blocked HMGA1 silencing-induced inhibition of colony formation, invasion, and migration (Figures 5D–F). Overall, these results indicated that HMGA1 could modulate autophagy and contribute to the proliferation, invasion, and migration of BC cells.

### MiR-221 Rescues the Impact of High-Mobility Group AT-Hook 1 Downregulation

**Figure 6:**
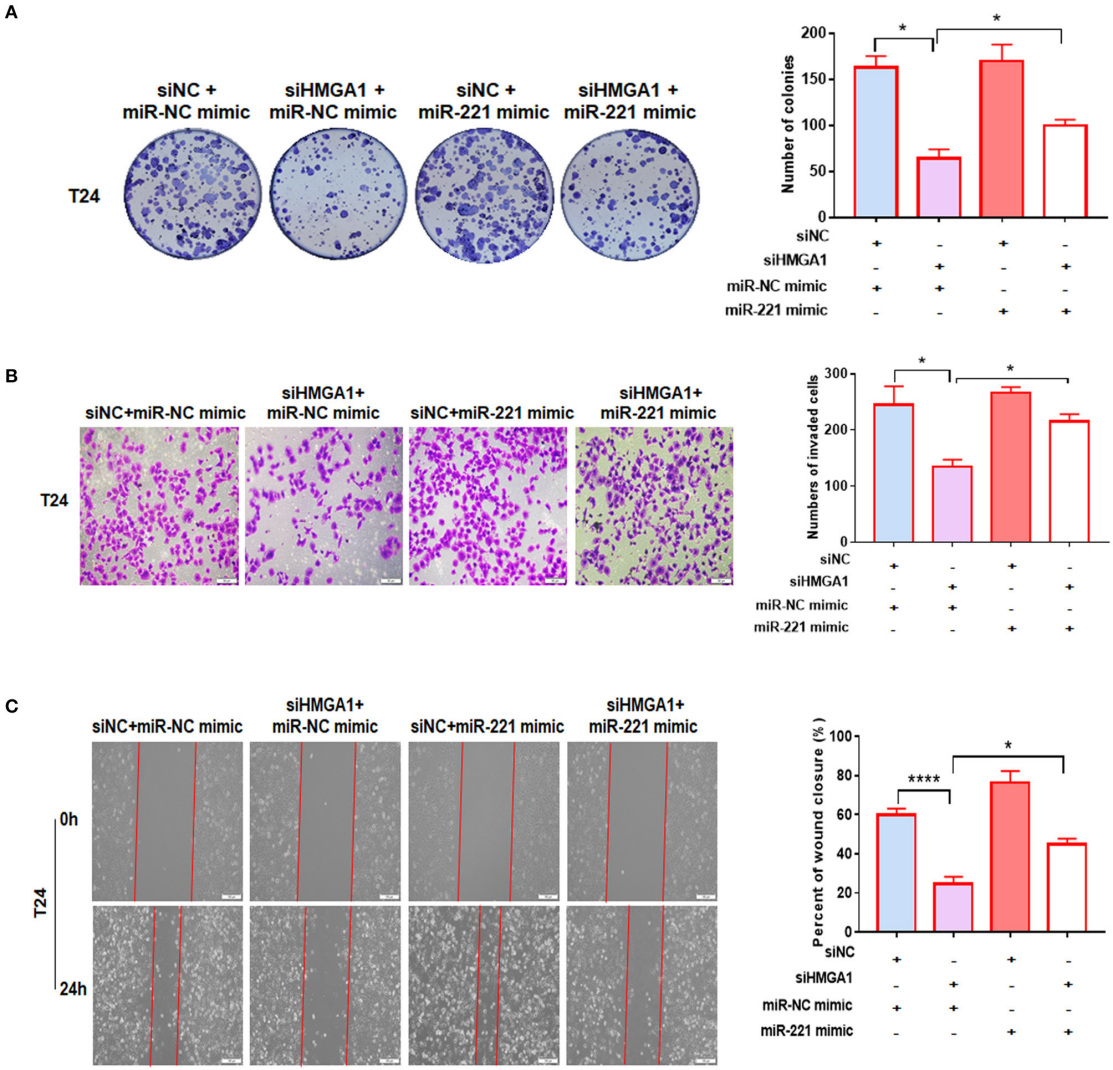
MiR-221 reversed the high-mobility group AT-hook 1 (HMGA1) silencing-induced decrease in proliferation, migration, and invasion in bladder cancer (BC) cells. **(A)** Colony formation assay indicated that HMGA1 silencing inhibited cell proliferation of BC cells, and the effect was abolished by miR-221 mimics. **(B)** Transwell invasion assay demonstrated that downregulation of HMGA1 decreased the invasive capacity of BC cells, and this effect was eliminated by miR-221 mimics. Scale bar, 50 μm. **(C)** Wound healing assay revealed that HMGA1 silencing diminished the migratory ability of BC cells, and this effect was abrogated by miR-221 mimics. Scale bar, 100 μm. *p < 0.05 and ****p < 0.0001.

We performed a colony formation assay, transwell invasion assay, and wound healing assay to further investigate the relationship between miR-221 and the effect induced by HMGA1 silencing. We found that miR-221 mimics could partly increase the efficiency of BC cell colony formation inhibited by the knockdown of HMGA1 (Figure 6A). Moreover, HMGA1 knockdown markedly inhibited cell invasion and migration, and miR-221 could rescue their invasive and migratory potential (Figures 6B,C).

### Downregulation of High-Mobility Group AT-Hook 1 Modulates Autophagy Partly by miR-221/TP53INP1 Axis

**Figure 7:**
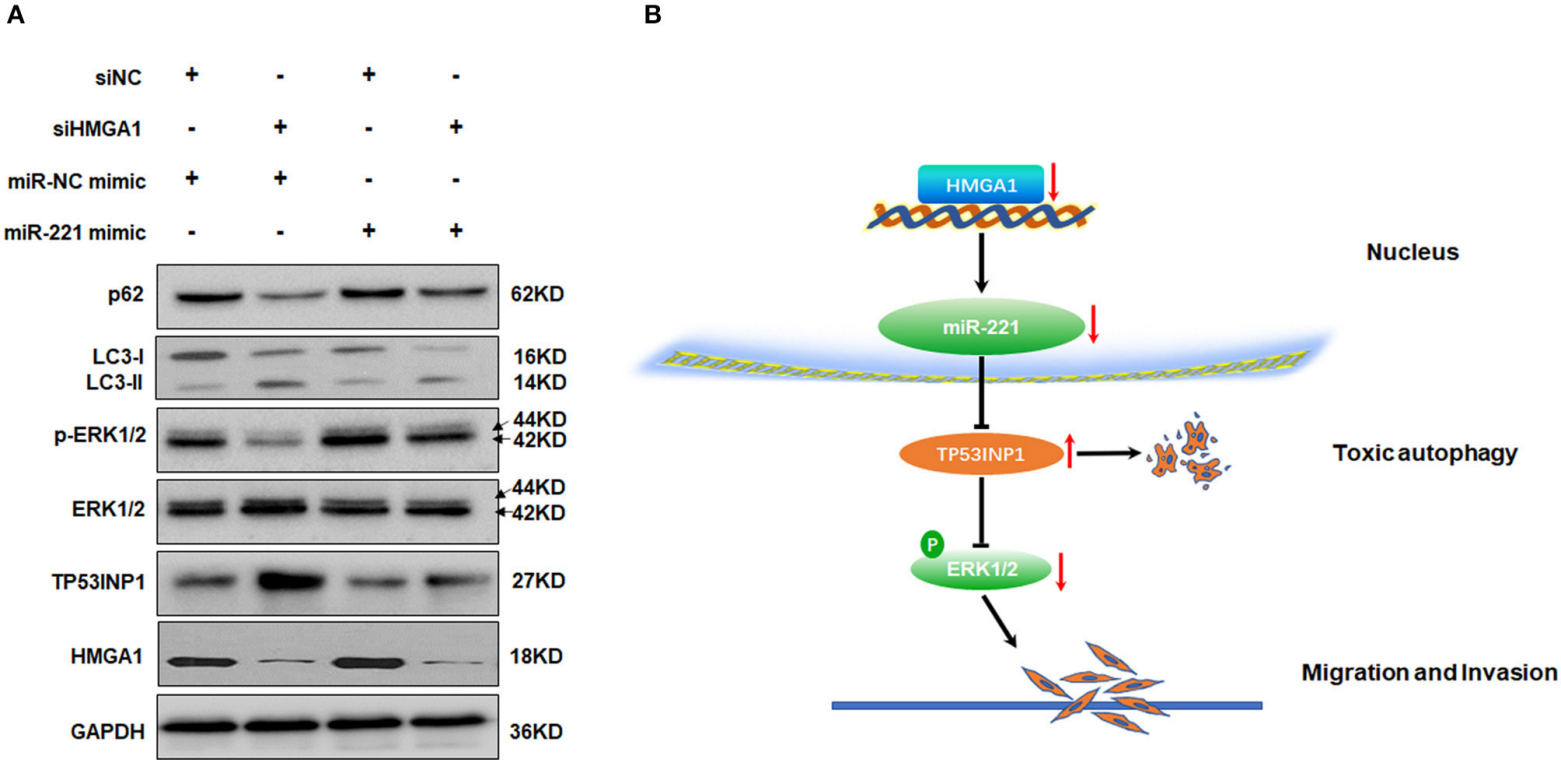
MiR-221 antagonized the downregulation of high-mobility group AT-hook 1 (HMGA1)-mediated autophagy and the proposed model of the role of HMGA1 in autophagy and bladder cancer (BC) migration and invasion. **(A)** Immunoblotting analysis of p62, LC3, TP53INP1, ERK1/2, phosphorylated-ERK1/2, and HMGA1 protein expression in T24 cells transfected with siRNA targeting HMGA1 (siHMGA1) and/or miR-221 mimic for 48 h. Glyceraldehyde-3-phosphate dehydrogenase (GAPDH) was used for normalization. **(B)** Downregulation of HMGA1 mediates reduction of MiR-221 which increases the level of autophagy by directly targeting TP53INP1, followed by inhibition of the extracellular signal-regulated kinase (ERK) pathway, and results in the suppression of migration and invasion in BC.

Given that miR-221 was regulated by HMGA1 and positively correlated with HMGA1 expression in BC, we hypothesized that the regulation of miR-221 may account for the effects on autophagy caused by HMGA1 silencing. Subsequent Western blot analyses showed that the downregulation of HMGA1 increased the LC3-II/LC3-I expression ratio and decreased the p62 expression in T24 cells, whereas the miR-221 mimic significantly reversed the effect of HMGA1 silencing (Figure 7A). Furthermore, the miR-221 mimic significantly antagonized the increase in TP53INP1 expression and reduction in ERK1/2 phosphorylation mediated by HMGA1 silencing (Figure 7A). Overall, these data suggested that the downregulation of HMGA1 modulated autophagy partly through the miR-221/TP53INP1 axis (Figure 7B).

## Discussion

Recent studies have indicated that miR-221 acts as an oncogene in the tumorigenesis and progression of various cancers (18, 34, 35). Our study revealed that high expression of miR-221 was correlated with poor prognosis of BC patients, and downregulation of miR-221 strongly impeded BC cell invasion and migration by inducing autophagy via modulating TP53INP1/p-ERK1/2 axis. The suppression of miR-221 prevented BC proliferation *in vivo*. Moreover, we found that miR-221 was regulated by HMGA1. HMGA1 silencing induced a reduction of miR-221 and autophagy and inhibited the proliferative, invasive, and migratory capacities of BC cells by loss-of-function experiments, and overexpression of miR-221 rescues the effect of HMGA1 downregulation. In these contexts, our study supports that miR-221 and HMGA1 are potential therapeutic targets for BC.

MiR-221 acts as an important regulator of autophagy balance and cardiac remodeling by modulating the p27/CDK2/mTOR axis, and miR-221 has been implicated as a therapeutic target in heart failure (27). Moreover, miR-221 is downregulated by the overexpression of *mda-7*/IL-24 in a cancer cell-specific manner, and downregulation of miR-221 can cause toxic autophagy and cancer cell-specific death (36). In this study, we confirmed that miR-221 acts as an oncogene in BC. Through gain- or loss-of-function studies, we found that the downregulation of miR-221 could promote autophagy in BC. In addition, TP53INP1, which acts as a tumor suppressor by inducing cell death via caspase-dependent autophagy (37), was identified as a target of miR-221 through luciferase reporter assay (Figure 2B). TP53INP1 knockdown could in part abrogate the effect of miR-221 suppression-induced autophagy and inhibition of cell invasion and migration (Figures 2C–E).

The activation of mitogen-activated protein kinase (MAPK)/ERK plays a critical role in tumor progression and invasion (38). TP53INP1 downregulation can activate the ERK pathway in a p73-dependent DUSP10 manner (31). We now show that TP53INP1 is a target of miR-221, and the overexpression of miR-221 can promote cancer cell migration and invasion through the TP53INP1/p-ERK1/2 axis.

MiRNAs have been shown to be regulated by the upstream transcription factors (39). However, the transcriptional regulation of miR-221 in BC has not been reported. MiR-221 and miR-222 are highly homologous miRNAs that are significantly overexpressed in many human cancers (12). MiR-222 was reported to be regulated by HMGA1 which can promote cell proliferation through binding to the promoter of miR-222 in lung cancer (15). Moreover, HMGA1 can accelerate migration/invasion in cervical cancer via regulating the transcription of miR-221/222 (18). Bioinformatics mining in TCGA dataset showed that miR-221 was positively correlated with the expression of HMGA1 in BC samples (Figure 4A). This positive correlation indicated that HMGA1 might regulate its expression in BC. This relationship has been confirmed by HMGA1 silencing which resulted in the downregulation of miR-221 and the upregulation of TP53INP1 (Figure 4B). HMGA1 silencing also resulted in the downregulation of miR-222 (data not shown). But TP53INP1 did not increase when the miR-222 inhibitor was transfected into T24 and 5637 BC cells (data not shown).

HMGA1 is reported to have critical roles in the tumorigenesis and progression of various cancers (13). However, the role of HMGA1 in BC remains unknown. We now demonstrate that HMGA1 is overexpressed in BC cell lines and tissues, and HMGA1 high expression was associated with poor prognosis in BC. Moreover, our study showed that HMGA1 silencing could induce autophagy but not apoptosis in BC cells. HMGA1 silencing could inhibit the proliferative, invasive, and migratory capacities of BC cells, and these effects are autophagy-dependent. The previous study reported that HMGA1 inhibits autophagy through negatively regulating ULK1 transcriptional activity (6). HMGA1 has also been demonstrated to activate phosphoinositide 3-kinase (PI3K)/AKT and mammalian target of rapamycin complex 1 (mTORC1) pathway (40), which strongly inhibits autophagy in cancer cells (41). We have shown here that HMGA1 is involved in autophagy through regulating the miR-221/TP53INP1 axis. As a transcriptional factor, HMGA1 may have dozens of targets. HMGA1 may modulate autophagy through other mechanisms. But our results revealed that HMGA1 silencing could downregulate the expression of miR-221 and upregulate the levels of TP53INP1, which could interact with LC3 and promote autophagy-dependent cell death (37). Furthermore, the overexpression of miR-221 could inhibit HMGA1 downregulation-induced autophagy and partly reinstate the biological effect of HMGA1 downregulation. Therefore, the miR-221/TP53INP1 axis is regulated by HMGA1, and HMGA1 plays a vital role in BC progression.

In summary, this study highlights MiR-221 is regulated by HMGA1 and can target TP53INP1/p-ERK1/2 axis to inhibit autophagy and promote migration and invasion in BC cells. MiR-221 and HMGA1 are correlated with poor patient survival in BC and are valuable therapeutic targets.

## Data Availability Statement

The datasets generated for this study are available on request to the corresponding author.

## Ethics Statement

The studies involving human participants were reviewed and approved by the first affiliated hospital of Nanchang university. The patients/participants provided their written informed consent to participate in this study. This animal study was reviewed and approved by the first affiliated hospital of Nanchang University.

## Author Contributions

XL, ZZ, and YW performed the experiments and generated data. XL, WD, KZ, YulL, XZ, LC, YuL, and AX analyzed data. TZ, GW, and BF designed the experiments. XL and BF wrote the manuscript. All authors reviewed and approved the manuscript.

## Conflict of Interest

The authors declare that the research was conducted in the absence of any commercial or financial relationships that could be construed as a potential conflict of interest.
