# Corrigendum: Downregulation of HMGA1 Mediates Autophagy and Inhibits Migration and Invasion in Bladder Cancer via miRNA-221/TP53INP1/p-ERK Axis

**DOI:** 10.3389/fonc.2020.01735

**Published:** 2020-09-04

**Authors:** Xiaoqiang Liu, Zhengtao Zhou, Yibing Wang, Ke Zhu, Wen Deng, Yulei Li, Xiaochen Zhou, Luyao Chen, Yu Li, An Xie, Tao Zeng, Gongxian Wang, Bin Fu

**Affiliations:** ^1^Department of Urology, The First Affiliated Hospital of Nanchang University, Nanchang, China; ^2^Jiangxi Institute of Urology, Nanchang, China; ^3^Department of Emergency, The Second Affiliated Hospital of Nanchang University, Nanchang, China; ^4^Department of Urology, The People's Hospital of Jiangxi Province, Nanchang, China

**Keywords:** high-mobility group AT-hook 1 (HMGA1), bladder cancer, autophagy, miR-221, TP53INP1

In the original article, there was a mistake in the legend for Figures 3D,E as published. “Scale bar, 100 μm” was erroneously written as “Scale bar, 50 μm.” The correct legend appears below.

In the original article, there were also mistakes in Figure 3E and Figure 5F as published. Due to carelessness, the picture for the 24 hour “Lenti-miR-NC + siATG5” group in Figure 3E was a duplication of the image for the 24 hour “Lenti-miR-NC + siNC” group. The picture of the 0 hour “siHMGA1 + siATG5” group in Figure 5F was an erroneous duplication of the “siNC + miR-NC mimic” group in Figure 6C. After carefully examining our original data, Figure 5F was erroneously placed. We found that the errors were caused by our carelessness in exporting the representative images and compiling these figures. The corrected Figure 3 and Figure 5 appear below.

**Figure 3:**
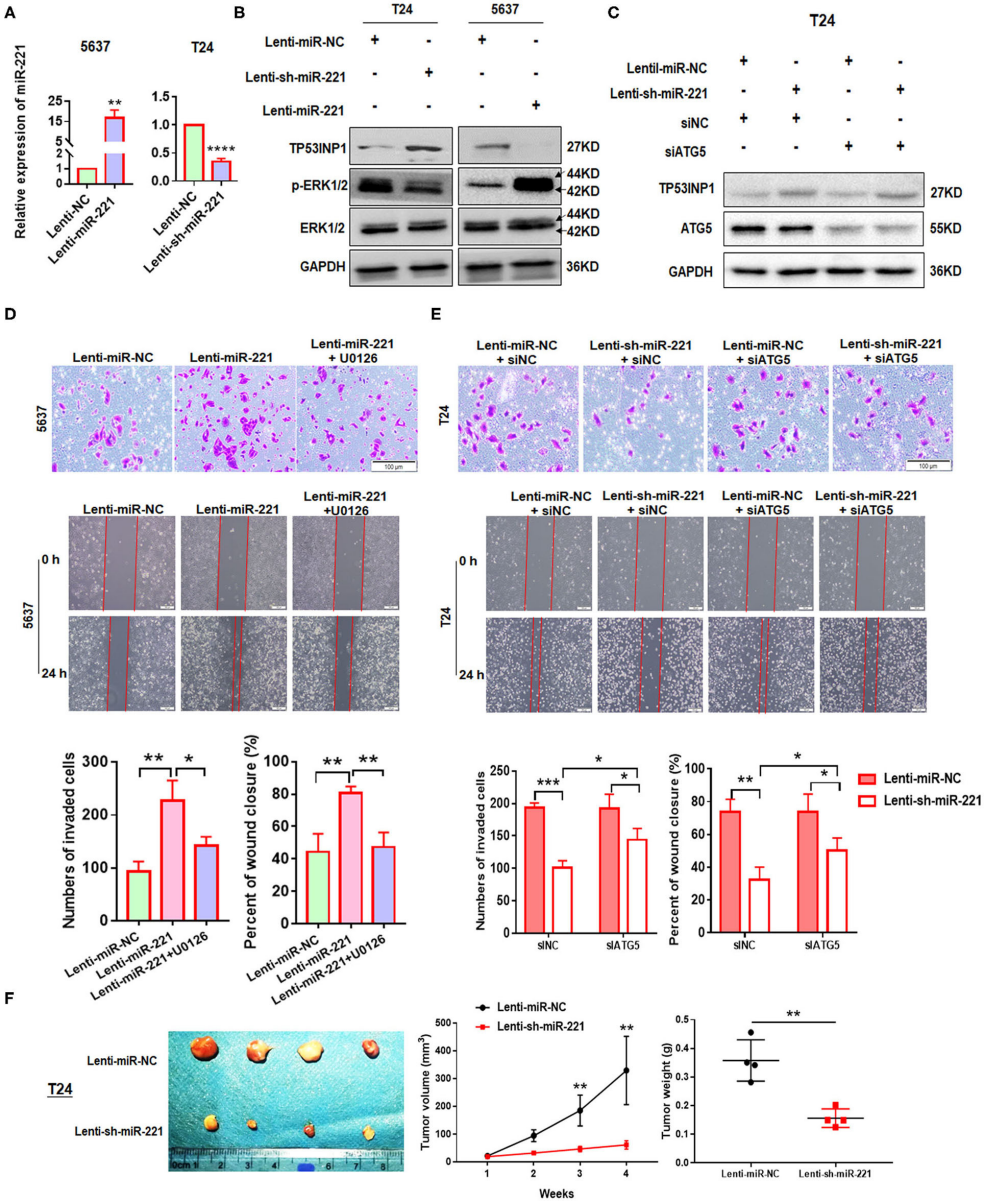
MiR-221 suppression-induced inhibition of invasion and migration is autophagy dependent. **(A)** Relative expression of miR-221 after overexpression and knockdown of miR-221 in 5637 and T24 cells detected by RT-qPCR. **(B)** Validation of the expression of TP53INP1, p-ERK1/2, and ERK1/2 using Western blot analysis. Glyceraldehyde-3-phosphate dehydrogenase (GAPDH) was detected as loading control. **(C)** T24 cells were transfected with Lenti-sh-miR-221 or siRNA targeting ATG5 (siATG5). **(D,E)** Transwell invasion assays and wound healing assay of 5637 cells treated with Lenti-miR-221 and ERK inhibitor U0126 and T24 cells treated with Lenti-sh-miR-221 or siRNA targeting ATG5. Scale bar, 100 μm. **(F)** For the *in vivo* analyses, 5 × 10^6^ T24-sh-miR-221 cells were injected subcutaneously into the posterior hip of nude mice. The mice were continuously observed for 30 days. Images of the tumors generated by T24-sh-miR-221 cells (*n* = 4). Tumor volume was monitored every week. Tumor weight was evaluated in T24-sh-miR-221 or miR-NC treated mice. *p < 0.05, **p < 0.01, ***p < 0.001, and ****p < 0.0001.

**Figure 5:**
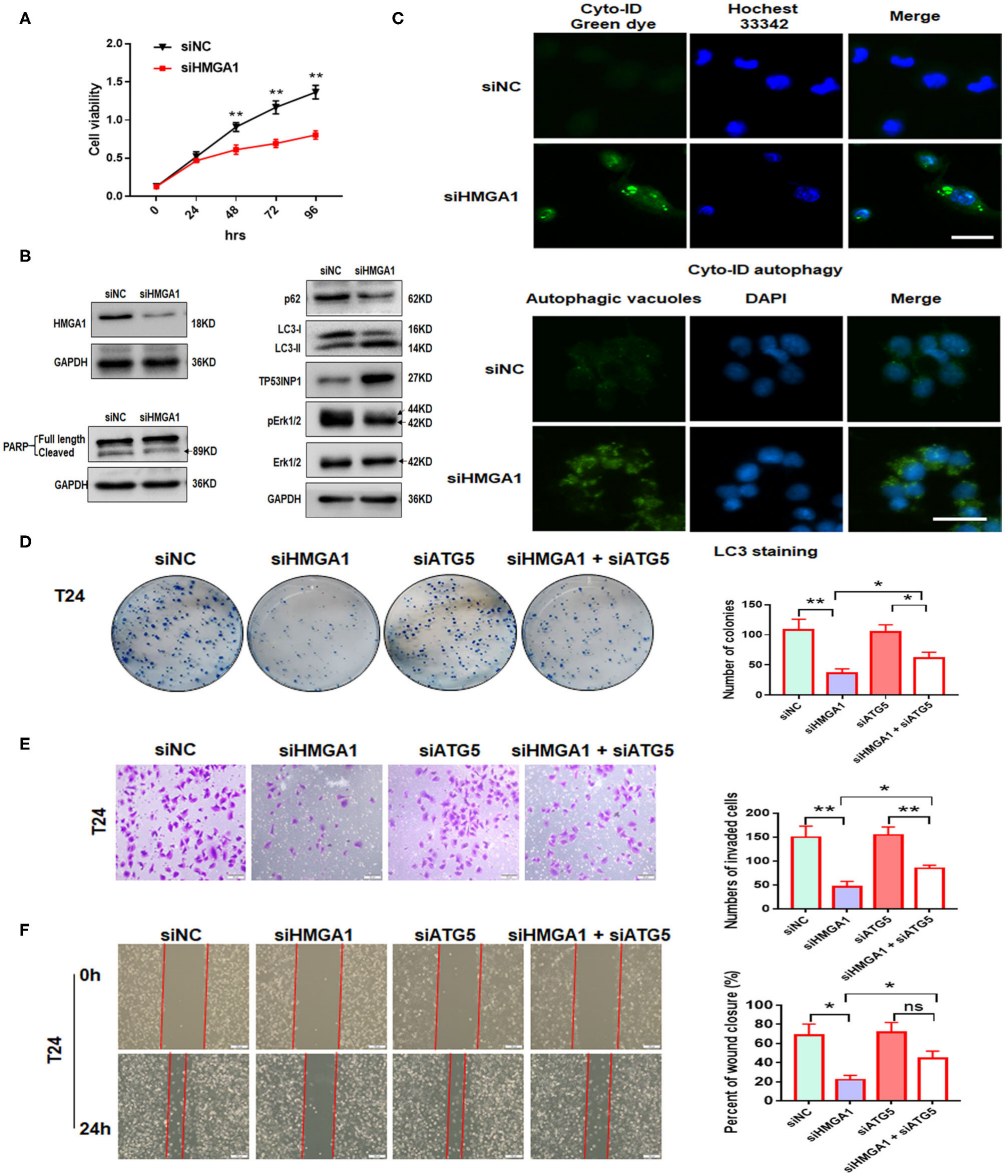
The effect of high-mobility group AT-hook 1 (HMGA1) knockdown on autophagy and growth, invasion, and migration of bladder cancer (BC) cells. **(A)** Cell Counting Kit-8 (CCK-8) cell viability assay of T24 cells treated with siHMGA1. **(B)** The protein expression of HMGA1, poly (ADP-ribose) polymerase (PARP), p62, LC3, TP53INP1, p-ERK1/2, and ERK1/2 in T24 cells transfected with HMGA1 siRNA. **(C)** T24 cells were transfected with HMGA1 siRNA for 48 h and then stained with Cyto-ID Autophagy Detection Kit and LC3 antibody. Scale bar, 50 μm. **(D)** Cell clone numbers were counted when HMGA1 or ATG5 was silenced in T24. **(E)** The effect of HMGA1 or ATG5 knockdown on the invasive ability of T24 cells as detected by transwell invasion assay. Scale bar, 50 μm. **(F)** The effect of HMGA1 or ATG5 silencing on cell migration of T24 cells performed by wound healing assay. Scale bar, 100 μm. *p < 0.05, **p < 0.01 and ns, no statistical significance.

The authors apologize for this error and state that this does not change the scientific conclusions of the article in any way. The original article has been updated.

